# Oxidative stress induction in woodworkers occupationally exposed to wood dust and formaldehyde

**DOI:** 10.1186/s12995-021-00293-4

**Published:** 2021-02-09

**Authors:** Federica Ghelli, Valeria Bellisario, Giulia Squillacioti, Elena Grignani, Giacomo Garzaro, Martina Buglisi, Enrico Bergamaschi, Roberto Bono

**Affiliations:** 1 Department of Public Health and Pediatrics, University of Turin, via Santena 5 bis, 10126 Turin, Italy; 2 Maugeri Scientific Clinical Institutes, Via Salvatore Maugeri, 10, 27100 Pavia, Italy; 3 Department of Public Health and Pediatrics, University of Turin, via Zuretti 29, 10126 Turin, Italy

**Keywords:** Occupational hygiene, Biomonitoring, Epidemiology, Formaldehyde, Wood dust

## Abstract

**Background:**

Many workers are exposed to wood dust (WD) and formaldehyde (FA), whose carcinogenic activity is supposed to be oxidative stress-mediated. This study aims to assess to what extent the occupational exposure to WD and FA, albeit within regulatory limits, could result in OS induction in a woodworkers’ population.

**Methods:**

The sample population consisted of 127 woodworkers from 4 factories and 111 unexposed controls. Individual exposure was assessed by personal air-samplers. Each participant enrolled in the study filled out a questionnaire and provided a urinary sample to quantify OS biomarkers, namely 15-F_2t_-IsoProstane (15-F_2t_-IsoP) and 7,8-dihydro-8-oxo-2′-deoxyguanosine (8-oxo-dGuo). The main confounding factor for OS, i.e. tobacco smoking exposure, was assessed by measuring cotinine in urine samples.

**Results:**

Woodworkers were exposed to significantly higher amounts of WD and FA as compared to controls (*p* < 0.001). Among OS biomarkers, 15-F_2t_-IsoP showed statistically significant higher values in woodworkers compared to controls (*p* = 0.004). A significant, positive correlation was observed between 15-F_2t_-IsoP and 8-oxo-dGuo (*p* = 0.005), cotinine (*p* = 0.05), FA (*p* < 0.001) and WD (*p* = 0.01); 8-oxo-dGuo was significantly correlated with cotinine (*p* = 0.001) and WD (*p* = 0.004). In addition, WD and FA were significantly correlated each other (*p* < 0.001).

**Conclusions:**

The study confirms that WD and FA may induce OS in woodworkers, and highlights that even the compliance with occupational exposure limits can result in measurable biological outcomes.

## Background

Wood dust (WD) is a complex mixture of cellulose (40–50%), polyose and lignin with a variable number of polar, non-polar and water-soluble compounds, but its exact formula depends on the species of tree processed [1, 2]. Over decades WD has become one of the most common occupational exposure scenarios, with millions of workers exposed worldwide [2, 3] and it has been associated with various adverse health impairments, such as dermatitis, allergic and non-allergic respiratory effects and cancer [4]. Thus, as early as 1995, the International Agency for Research on Cancer (IARC) classified WD as “carcinogenic to humans (Group 1)”, considering the increasing risk in subjects exposed to WD in developing cancers of the nasal cavities and paranasal sinuses [2]. Nevertheless, non-malignant respiratory effects could also occur, including, among the others, upper and lower respiratory tract symptoms and inflammation and occupational asthma [5]. In vitro exposure to fine particulate matter may lead to the increased production of Radical Oxygen Species (ROS) [6]. Oxidative Stress (OS), i.e. the imbalance between generation of oxidant compounds and of antioxidant defences induced by ROS [7], seems a likely fundamental mechanism involved in WD toxicity in airway cells. Particularly, the short-term exposure to pine, birch and oak dusts was found to induce a significant increase in the production of ROS in human bronchial epithelial cells with a subsequent increase of the caspase-3 activity [6].

Wood industry intensively uses also chemicals, such as formaldehyde-based resins, which are used in wood-based panels and dyes [8]; as a result, high concentrations of formaldehyde (FA) have been measured in these workplaces [9]. The last update by IARC confirmed FA as “carcinogenic to humans (Group 1)” because it may promote the nasopharyngeal cancer and leukaemia, whereas evidence for association with sinonasal cancer was limited [10]. FA is a potent trigger of inflammation in the lower airways, and exposure to this chemical may increase the production of ROS, leading to the respiratory system damaging and promoting structural and enzymatic changes in target organs [11].

Exposure over an extended period of time to toxic environmental pollutants could lead to significant toxic insults, even at low concentrations [12]. OS may be thus one of the plausible biological mechanisms behind WD and FA toxicity [3, 13] possibly involved in the inflammation processes, which have been associated to cancer progression [14]. In fact, inflammation and OS are closely related pathophysiological events [15]: OS can trigger redox-sensitive pathways and lead to different biological processes like inflammation and cell death [16]. Among OS biomarkers, 15-F_2t_-IsoProstane (15-F_2t_-IsoP) is a prostaglandin-like molecule generated by the free radical-induced peroxidation of arachidonic acid [17]. It is ubiquitous in the body, chemically stable in biological fluids and widely considered a reliable, sensitive and specific non-invasive biomarker of in vivo systemic OS [17, 18]. Recently it has been speculated that 15-F_2t_-IsoP excretion is associated with 7,8-dihydro-8-oxo-2′-deoxyguanosine (8-oxo-dGuo or 8-oxo-dG) [19, 20], a guanine modification induced by free radicals and adopted as a non-invasive biomarker of OS [21]. The accumulation of such damages can lead to genetic information changes, and, consequently, to mutagenesis and cells apoptosis [22]. The urinary concentration of this biomarker reflects the equilibrium between its production and repair in both DNA and the nucleotide pool [23].

*Wultsch* et al. found elevated concentrations of biomarkers indicative for inflammation and OS such as C-reactive proteins and MDA in woodworkers, with MDA levels significantly higher than those measured in the control group and exceeding the levels found in a reference healthy population [24]. In a recent study, *Bono* et al. found an increase in urinary 15-F_2t_-IsoP levels in woodworkers compared to a control group [25].

In this work we want to further explore the relationship between the exposure scenario distinctive of wood factories and the potential increase of OS in woodworkers. We decided to use a more detailed approach, recruiting exposed workers in companies performing different types of industrial processes, by measuring both the personal exposure to aspecific WD and FA and OS biomarkers (15-F_2t_-IsoP and 8-oxo-dGuo), which reflect the result of different metabolic pathways.

Therefore, the purpose of this work was to clarify to what extent the occupational exposure to WD and FA, albeit in compliance with regulatory limits, could result in OS, namely the urinary levels of 15-F_2t_-IsoP and 8-oxo-dG, in a woodworkers’ population. The assumption is that the everyday occupational exposure to WD and FA, even at low doses, could lead to an increase in OS.

## Materials and methods

### Epidemiological sample

The study population consisted of 238 volunteers, including 127 exposed and 111 unexposed controls.

The exposure group included woodworkers recruited in 4 factories located in Piedmont (Italy), operating in different sectors of the wood industry. Two plywood factories (A-B) were specialised in the production of panels employed in different sectors, such as furniture and marine industry. The wood veneers employed in these factories are mainly softwood and poplar and the manufacturing processes include the processing of raw material, veneer cutting, drying, gluing, composition, pressing, squaring and sanding. Factories C and D were a company specialised in door production (C) and a furniture factory, respectively. In these facilities, many wood veneers were employed, mainly softwood, and the woodwork can be outlined in milling, carving, shaping and sanding and painting. Workers were in compliance with the use of the personal and environmental protective equipment (including respiratory protective equipments, when necessary), according to the regulations in force.

However, given that our aim was to highlight the induction of OS in wood workers exposed also to FA as co-determinant, the different quality of the exposures of wood workers was useful for the achievement of biological purpose.

The control group involved workers of secondary and tertiary sectors not occupationally exposed to WD. Sampling was carried out over the period between 2016 and 2017. Every participant filled out a questionnaire, provided a urinary sample and wore two personal environmental samplers, a passive one for FA and an active one for WD. The study was approved by the University of Turin ethics committee and the study is in line with ethical standards reported in The Code of Ethics of the World Medical Association (Declaration of Helsinki, 2013).

### Questionnaire

Every participant filled out a questionnaire providing information about personal data including height (expressed in m) and weight (expressed in kg), used for calculating Body Mass Index (B.M.I.) (kg/m^2^). Additional details on working conditions (years of working, presence of ventilation system, annual check of ventilatory flow test, employment of Personal Protective Equipment (PPE), medical *status* (with a specific focus on respiratory disorders and allergies) and lifestyle were provided.

### Environmental exposure

#### Dust

The inhalable fraction of dust was collected by SKC Button Aerosol sampler equipped with PVC fiber filters (0.5 μm, Ø 25 mm, Whatman) and a pump (Gilian 5000, Sensidyne, USA) operating with a flow rate of 4 l/minute. The active personal samplers were worn by participants with the size selector clipped near the breathing zone during an 8 h-working shift.

The WD concentration was determined by gravimetric analysis. Before and after each sampling, filters were dehydrated in a sealed bell with silica gel at 180 °C and kept in the dark for at least 72 h. Then clean filters were stored at 4 °C until sampling, as well as the dusty ones until the second dehydration. The weighing was performed by an analytical scale with a 0.00001 g sensitivity. Every filter was weighed 3 times and we considered the average value. A control filter (not used in samplings) was included in every dehydration treatment, in order to allow constant monitoring of the cooling-weighing system. The formula used to quantify the WD [mg] is WD = C/(L/1000), where C is the difference between the average value of dusty filter weight [mg] and clean filter weight [mg] and L are the litres sampled by the pump.

#### FA

Individual exposure to indoor FA was collected during an 8-h working shift using radial diffusive passive samplers (Radiello®) clipped near the breathing zone of each subjects. Samplers were equipped with a specific cartridge filled with 2,4-dinitrophenylhydrazine (2,4-DNPH) coated (Florisil®). The 2,4-DNPH reacts with FA yielding 2,4-dinitrophenylhydrazone, which can be subsequently extracted with acetonitrile and analysed by reverse phase HPLC and UV detection, according to manufacturer’s instructions. The analyses were performed in the laboratories of *Fondazione Salvatore Maugeri* (Padua, Italy).

### Biological assessment

Four urine aliquots were collected from each participant to quantify the concentration of creatinine (crea), 15-F_2t_-IsoP, 8-oxo-dGuo and cotinine. Urine spot samples were collected at the beginning of the third working day shift of the week, as representatives of the week exposure. Aliquots were stored at − 80 °C prior to analysing.

#### Creatinine

Urinary creatinine was determined by the kinetic Jaffé procedure [26] to normalise the urinary excretion rate of 15-F_2t_-IsoP, 8-oxo-dGuo and cotinine.

#### 15-F_2t_-IsoP

The urinary 15-F_2t_-IsoP concentration was measured by a competitive enzyme-linked immunoassay (ELISA) performed with a specific microplate kit (Oxford, MI, USA), according to manufacturer’s instructions. The 15-F_2t_-IsoP concentrations were normalised to the individual creatinine levels [25].

#### 8-oxo-dGuo

The urinary 8-oxo-dGuo was determined by Acquity UPLC system coupled with a triple quadrupole Waters TQD mass spectrometer (Waters, Milford, MA, USA). The method was validated according to EMEA bioanalytical method guideline (EMEA 2011). Briefly, after thawing, internal standard ([15 N5]8-oxo-7,8-dihydro-2′-deoxyguanosine) is spiked to urine samples (1.0 ml); after mixing and centrifugation, samples were diluted in 100 mM formic acid solution (1:4), filtered using 0.2 μm Acrodisc Syringe Filter and injected onto UPLC system. Chromatographic separation was performed on a UPLC BEH C18 column (2.1 × 100 mm, 1.7 μm) maintained at 40 °C and by gradient elution with a mixture containing variable proportions of 20 mM formic acid solution and methanol; flow rate was delivered at 0.5 ml/min, retention time of 8-oxo-dGuo and its internal standard was 1.8 ± 0.01 min. Injection volume was 7.5 μl. For the mass spectrometer detection, electrospray was operated in positive ion mode and the acquisition was performed in MRM (Multi Reaction Monitor); in particular, for 8-oxo-dGuo: m/z 284.0 → 167.9 for quantification (CV 18, CE 15) and m/z 284.0 → 116.9 for confirmation (CV 18, CE 19); for 15 N5 8-oxo-dGuo: m/z 289.0 → 173.0 (CV 18, CE 15). The method is accurate and precise, and provides a broad linear concentration range of 3–100 nM using Sigma synthetic urine. LLOQ was 1.5 nM (Guideline on bioanalytical method validation. 21 July 2011; EMEA/CHMP/EWP/192217/2009 Rev. 1 Corr. 2**; Committee for Medicinal Products for Human Use (CHMP)). The 8-oxo-dGuo concentrations were normalized to the creatinine levels. The analyses were performed in the laboratories of “Fondazione Salvatore Maugeri” (Padua, Italy).

#### Cotinine

The urinary cotinine concentration was measured using the cotinine ELISA kit (KA0930 Abnova, Taiwan), according to manufacturer’s instructions, as previously described [27]. The declared limit of detection is 1 ng/ml. The cotinine concentrations were normalized to the creatinine levels [28].

### Statistical analysis

Differences among groups were tested using Chi-square (Χ^2^) test for categorical variables (residence, smoking, working years, wheezing, asthma-like symptoms, allergies and eczema) and Wilcoxon test for continue variables (age, B.M.I. and cotinine), as appropriate. The Shapiro-Wilk test was performed to assess the normality of the distribution. For variables with a non-normal distribution a logarithmic transformation was performed. Non-parametric correlations were assessed by Spearman’s test to investigate the correlation between OS biomarkers (15-F_2t_-IsoP and 8-oxo-dG), tobacco smoking exposure (cotinine) and environmental variables (WD and FA concentrations). Multiple Linear Regression (MLR) models were used to evaluate the association between environmental variables (WD and FA), tobacco smoke exposure and OS biomarkers (15-F_2t_-IsoP and 8-oxo-dGuo). The MLR analyses were adjusted for cotinine, exposure to WD, ventilation, formaldehyde, residence, and age. Non-parametric comparisons of biological and environmental variables between exposed and control groups were assessed by Mann-Whitney U test. The Kruskal-Wallis test was performed to compare data concerning the different types of exposures found in the 4 factories and the control group. Statistical analyses were performed with SPSS Statistics 25 (IBM SPSS Statistics, New York, NY) and RStudio (RStudio Desktop 1.2.5042, RStudio Inc.).

## Results

Table 1 shows the demographic characteristics of the sample population grouped by exposure to WD. In the study population (*n* = 238), 67.6% were males.

**Table 1:** Demographics of the sampling population presented as exposed and control groups

		Exposed ***n*** = 127 (100%)	Controls ***n*** = 111 (100%)	***p***-value
Residence	Countryside	117 (92)	29 (26)	< 0.001
	Suburb	10 (8)	41 (37)	
	City	0 (0)	41 (37)	
Smoking	Yes	48 (38)	28 (25)	0.05
	No	79 (62)	83 (75)	
Working years	0-5	35 (28)	41 (37)	0.14
	5–10	21 (16)	22 (20)	
	> 10	71 (56)	48 (43)	
Wheezing	Yes	18 (14)	20 (18)	0.53
	No	109 (86)	91 (82)	
Asthma-like symptoms	Yes	8 (6)	15 (14)	0.10
	No	119 (94)	96 (86)	
Allergies	Yes	7 (6)	11 (10)	0.3
	No	120 (94)	100 (90)	
Eczema	Yes	29 (23)	26 (23)	1
	No	98 (77)	85 (77)	
Age [years]		44 (16)	40 (16)	0.020
B.M.I. [kg/m^2^]		23.5 (3.6)	23 (4.4)	0.353
Cotinine [ng/mg crea]		12.84 (42.47)	3.53 (18.29)	< 0.001

Differences between exposed and controls were tested using Χ^2^ and Wilcoxon tests for categorical and continue variables, respectively. Age, B.M.I. and cotinine are reported as Median value (IQR*)*

Table 2 reports the median values of biological and environmental parameters, in exposed subjects, according to the four factories where woodworkers were enrolled and in the control group. The Kruskal-Wallis test performed to compare data concerning the different types of exposures found in the 4 factories and the control group revealed significant differences in WD and FA concentrations and in 15-F2t-IsoP levels, while no difference was found in the amount of urinary 8-oxo-dGuo.

**Table 2:** Biological and environmental variables measured in exposed and control subjects express as *Median value (IQR)*

	Overall exposed	Factory A	Factory B	Factory C	Factory D	Controls	KW test
	*n* = 127	*n* = 38	*n* = 30	*n* = 46	*n* = 13	*n* = 111	*p-value*
WD [mg/m^3^]	0.34 (0.25)	0.39 (0.22)	0.33 (0.21)	0.22 (0.16)	0.45 (0.30)	0.04 (0.03)	p < 0.001
FA [μg/m^3^]	48.4 (68.3)	81.6 (68.2)	132.9 (125.3)	34.5 (9.1)	26.7 (4.2)	13.7 (15.0)	p < 0.001
15-F_2t_-IsoP [ng/mg crea]	3.29 (3.31)	3.66 (3.00)	3.37 (9.49)	3.37 (2.89)	2.78 (1.25)	2.51 (2.37)	p < 0.001
8-oxo-dGuo [μmol/mol crea]	1.02 (0.57)	1.00 (0.61)	0.95 (0.39)	1.15 (0.54)	0.98 (0.44)	1.15 (0.76)	*p* = 0.33

We performed non-parametric correlations (Spearman) on the whole sample, founding a significant correlation between WD and FA concentrations (Spearman’s rho = 0.35, *p* < 0.001) and between 15-F_2t_-IsoP and FA (rho = 0.63, *p* < 0.001), WD (rho = 0.16, *p* = 0.01), cotinine (rho = 0.13, *p* = 0.05), and 8-oxo-dGuo (rho = 0.18, *p* = 0.005). Conversely, 8-oxo-dGuo was positively and significantly correlated only with cotinine (rho = 0.223, *p* = 0.001) and WD (rho = 0.19, *p* = 0.004).

**Fig. 1:**
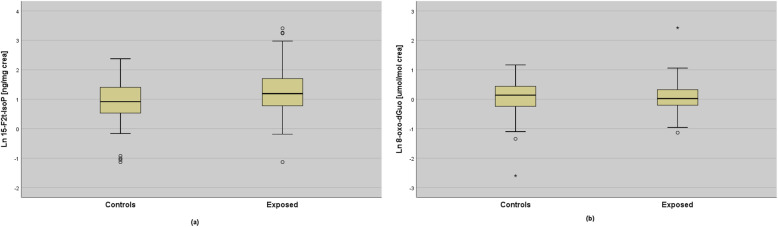
15-F_2t_-IsoP (**a**) and 8-oxo-dGuo (**b**) (ln-transformed data) between exposed and control groups

Non-parametric analyses (Mann-Whitney U test) between woodworkers and controls confirmed a higher exposure to WD and FA (*p* < 0.001) in woodworkers (*p* < 0.001). Among OS biomarkers, only 15-F_2t_-IsoP was higher in exposed workers as compared to controls (*p* = 0.004) (Fig. 1).

**Fig. 2:**
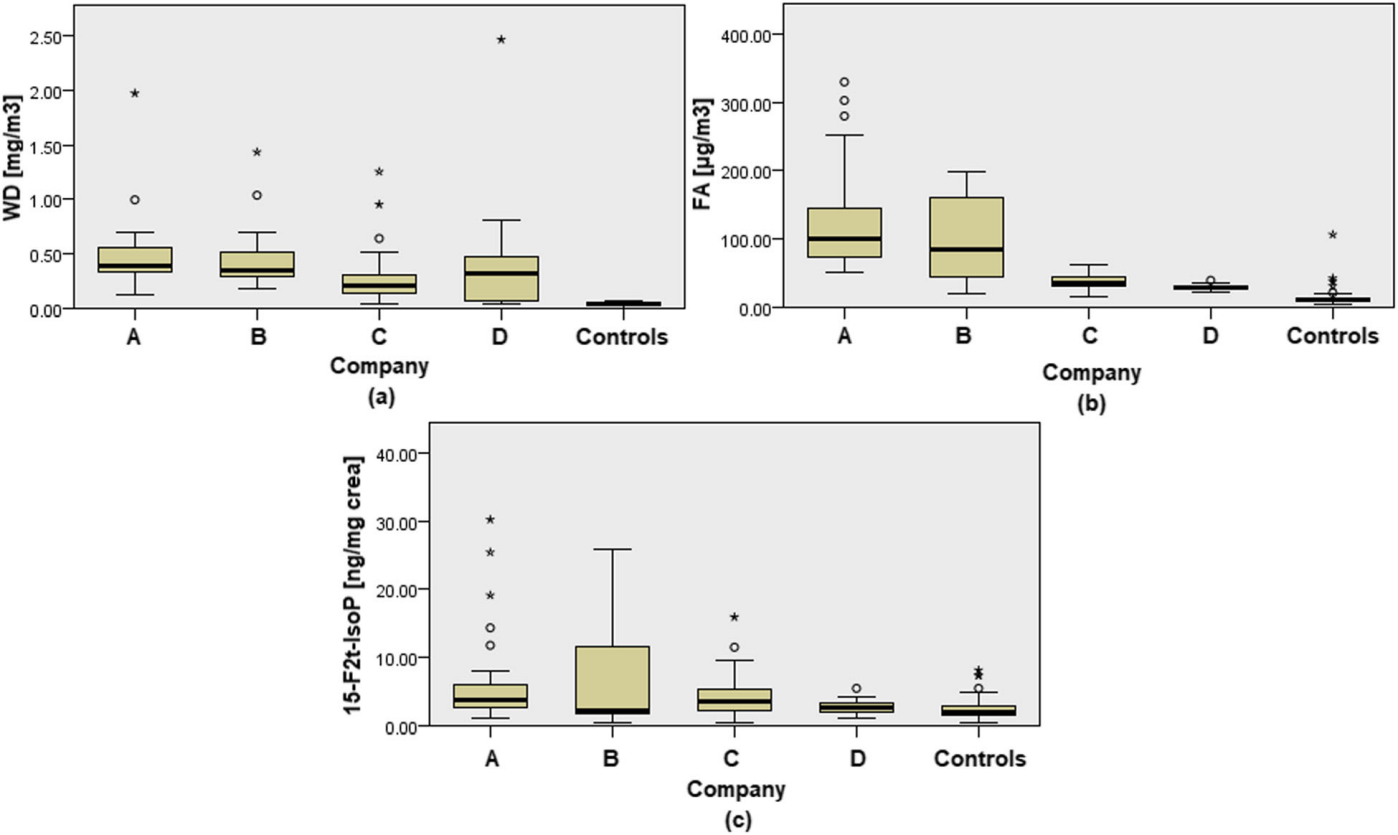
WD (**a**), FA (**b**) and 15-F_2t_-IsoP (**c**) concentrations among exposed workers and controls

We further tested the same hypotheses by grouping the exposed subjects according to the four sampling sites. As expected, controls were exposed to lower WD concentrations than workers from all considered companies (KW test, *p* < 0.001) and, among the four companies, A and B showed significantly higher concentration levels than C (pairwise comparison, *p* = 0.004 and *p* = 0.029, respectively) (Fig. 2a).

Concerning FA exposure, controls had lower levels than workers (KW test *p* < 0.001; pairwise comparison was significant between controls and A, B and C companies (*p* < 0.001 for all tested differences). FA concentrations measured in C and D were lower than those measured in A (*p* < 0.001 for both) and in B (*p* = 0.017 and *p* = 0.001, respectively) (Fig. 2b). Concerning OS biomarkers, instead, only workers employed in companies A and C turned out to have 15-F_2t_-IsoP levels higher than the control group (KW test *p* < 0.001; *p* = 0.001 and *p* = 0.002, respectively) (Fig. 2c).

MLR models with ln-transformed dependent variables, as described in Table 3, revealed that both 15-F_2t_-IsoP and 8-oxo-dGuo levels were positively influenced in a significant way by cotinine and FA concentration. In addition, the 8-oxo-dGuo level was influenced also by the exposure to WD.

**Table 3:** Results of the MLR analysis for 15-F_2t_-IsoP and 8-oxo-dGuo. The model was adjusted by cotinine, exposure to WD, ventilation, formaldehyde, residence, and age

	Exp (b)	95% C.I.	***p***-value
**15-F**_**2t**_**-IsoP**			
Cotinine	1.073	1.020–1.129	0.007
FA	1.527	1.122–2.078	0.007
**8-oxo-dGuo**			
Cotinine	1.165	1.063–1.277	0.001
FA	0.470	0.282–0.783	0.004
Exposure to WD	1.945	1.096–3.451	0.023

## Discussion

The aim of this study was to evaluate the possible effect of the combined exposure to WD and FA, both carcinogenic compounds, on OS biomarkers in woodworkers employed in different types of industrial processes. Although the harmful effects of the exposure to WD and FA have been known for a long time, only a few studies in literature focused on oxidative *status* alterations in woodworkers.

We investigated the molecular outcomes of this distinctive kind of exposure, because OS may be one of the mechanisms through which both these compounds could express their activity. Since OS is a complex phenomenon that can reveal itself through multiple pathways [29], we analysed the outcomes of two different biomarkers: 15-F_2t_-IsoP, a measure of lipid peroxidation, and 8-oxo-dGuo, a biomarker of DNA repair following oxidative damage.

Such a biomarker-based approach has been used to evaluate the environmental and occupational exposure to xenobiotics: the rate of DNA and lipid oxidation is known to be influenced, indeed, mainly by environmental factors [30]. This study has been carried out in woodworkers because this is a population known to be exposed to relevant amounts of WD and FA. FA is a quite common pollutant in wood industries, since it is a constituent of both plant organisms [31] and glues, resins and paints employed in the production of manufactures. This consideration could explain also the positive correlation observed between the two main agents under investigation. WD and FA concentrations detected in this study, however, were nearby, and generally lower than both occupational exposure limits adopted by EU. In particular, the WD average exposure measured in woodworkers was 0.41 mg/m^3^, lower than the threshold limit in force at the time of sampling. Indeed, the Italian law recommended a threshold exposure limit for WD of 5 mg/m^3^ (Legislative Decree No 81/2008) until 2020, when, in accordance with the European Directive Decree No 2017/2398, a new transitory limit of 3 mg/m^3^ has entered into force until 17 January 2023. The limit will drop further to 2 mg/m^3^ [25] thereafter. Also for FA, we found an average exposure level (76 μg/m^3^) much lower than the “Time Weighted Average” (average concentration for 8-h working day to which almost all workers can be exposed, repeatedly without suffering harmful effects on health) TLV-TWA of 0.1 ppm (i.e. 0.12 mg/m^3^) recommended by the American Conference of Governmental Industrial Hygienists (ACGIH) [32].

Nevertheless, our study reveals an alteration in the woodworker’s oxidative *status*: we found a significant increase in 15-F_2t_-IsoP, in accordance with a recent study performed in wood industries in Tuscany Region (Italy) [25], and a positive correlation between this biomarker level and the concentration of both WD and FA. On the contrary, we did not find any relevant differences concerning 8-oxo-dGuo.

Our results can be explained considering the different pathophysiological meaning of the two biomarkers and the different effects they reflect. 15-F_2t_-IsoP, unlike 8-oxo-dGuo, is not a marker of immediate effect, but it reflects the exposure to pollutants that occurred at least 3–4 weeks before sampling [33]. The possible explanation may be that the damage of lipids is not repaired, rather the lesions accumulated allowing the detection of longer exposures before sampling, while for genomic injuries the DNA repair mechanisms remove the damage to DNA shortly after it occurs [33]. Moreover, Bono et al. reported that M_1_dG, a major endogenous peroxidation-derived DNA adduct [34], can be detected as a result of FA exposures higher than 66 μg/m^3^ [35]: the low FA concentrations found in the current study, may not be enough to induce a measurable DNA oxidative damage, explaining, thus, the absence of correlation between FA and 8-oxo-dGuo.

A positive correlation between 15-F_2t_-IsoP and 8-oxo-dGuo was observed in the present study, even though contrasting reports can be found in literature [36–38].

Our data confirmed, once more, the role of tobacco smoking in OS induction as a positive correlation between urinary cotinine concentration and both 15-F_2t_-IsoP and 8-oxo-dGuo levels was highlighted, in accordance with previous reports [20, 39, 40]. In the MLR models, cotinine and FA were significant predictors of the variation of both 15-F_2t_-IsoP and 8-oxo-dGuo, straightening the role of both environmental exposure and tobacco smoking in OS induction. In this context, we have to point out that in Italy the smoking ban is in force in every workplace since 2008 (D.Lgs. 81/08), and not only where there is a risk of fire. Therefore, it is very unlikely that there could have been any kind of formaldehyde contamination due to second-hand smoke during the formaldehyde personal sampling.

The comparison among various types of wood industries allowed highlighting different exposure scenarios. All the companies showed a significantly higher WD concentration in comparison with the control group and the highest mean value was found in the furniture factory, even though a high variability was observed. Plywood industries workers resulted to be exposed to high concentration of both WD and FA, while the lowest mean dust level was detected in the doors factory. These differences could be explained considering different tasks performed by workers in the industries belonging to the wood sector. The different processes are known to produce particles with different size: smaller particles are associated with sanding, while larger particles with planning and shaping processes [41]. Concerning FA, the lower mean concentrations were found in the furniture industry and in the doors factory, among which no significant differences were found. The furniture manufacture turned out to be the only one where the measured FA level is not significantly different from that of the control group. In that context, the difference of processes might explain the variability among companies. In a FA exposure evaluation of Italian workplaces, the male workers potentially exposed to FA in wood-related mansions at higher risk were in the sector of “manufacture of veneer sheets, plywood, lamin-board, particle board, other panels and boards” [9].

The biological outcome seems to reflect the differences in exposure scenarios, even though the interpretation should be conducted carefully. The higher values were found in the two plywood industries and the lowest in the furniture manufactory. Nevertheless, the 15-F_2t_-IsoP levels found in the control group turned out to be significantly lower than the concentrations found in one of the plywood industries and in the doors factory. These data suggest that OS is the result of the exposure to a wide range of environmental and lifestyle factors, as well as of physio-pathological processes and aging, not investigated in this study.

The main weaknesses of the current study are the cross-sectional design, which prevents the possibility of causal inferences, and the fact that we did not consider all the possible confounding factors, which could also modulate in varying degrees these non-specific biomarkers, focusing only on key exposures of woodworkers. Moreover, in the wood sector several times companies work on commission: this element may affect the type of wood processed and, in turn, create variability in workers’ exposure. The heterogeneity of the WD produced in the different selected factories, but also in the same company, in different moments could affect the biological responses and thus, this issue still needs to be investigated. Longitudinal studies should be performed in order to confirm the relationships observed between the environmental exposure and the biological outcome in the wood sector. The main strength, instead, consist in the quantification of two different OS biomarkers in order to deepen the wide outcome range that could be related to WD and FA exposure. The main novelty of this research lies in the evaluation of biological data based on personal exposure measurement to WD and FA: the measurement of these two environmental factors with personal sampler allowed relating the oxidative *status* alteration with the pollutant concentration that could be found in the workers’ breathing zone.

## Conclusions

The study confirms that exposure to WD and FA can increase the level of endogenous metabolites reflecting OS, even though at exposure level below the currently adopted OELs.

Hence, the importance not only of the compliance with safety standards but also of the regular updating of these regulations based on the latest knowledge in order to protect the workers’ health. Not least, this study confirms the fundamental role of the preventive measures in order to protect the workers’ health, since even very low exposure levels turn out to be able to induce measurable biological alterations. These findings deserve further investigations to assess the relevance of different sources of FA in OS induction.

## Data Availability

The datasets used and/or analyzed during the current study are available from the corresponding author on reasonable request.

## Abbreviations

WD: Wood dust
IARC: International Agency for Research on Cancer
ROS: Radical oxygen species
OS: Oxidative stress
FA: Formaldehyde
15-F_2t_-IsoP: 15-F_2t_-IsoProstane
8-oxo-dGuo: 7,8-dihydro-8-oxo-2′-deoxyguanosine
B.M.I.: Body Mass Index
PPE: Personal Protective Equipment
PVC: polyvinyl chloride
UPLC: Ultra performance liquid chromatography
LLOQ: Lower limit of quantification
ELISA: Enzyme-linked immunoassay
MLR: Multiple linear regression
TLV-TWA: Threshold Limit Value – Time-Weighted Average
ACGIH: American Conference of Governmental Industrial Hygienists
M_1_dG: 3-(2-deoxy-β-D-erythro-pentafuranosyl) pyrimido [1,2-α]purin-10(3H)-one dG
OELs: Occupational exposure limits
